# A proposed staging system for chronic symptomatic pilonidal sinus disease and results in patients treated with stage-based approach

**DOI:** 10.1186/s12893-016-0134-5

**Published:** 2016-04-16

**Authors:** Ali Guner, Arif B. Cekic, Aydin Boz, Serdar Turkyilmaz, Uzer Kucuktulu

**Affiliations:** Department of General Surgery, Karadeniz Technical University, College of Medicine, Farabi Hospital, 61080 Trabzon, Turkey; Department of General Surgery, Trabzon Kanuni Training and Research Hospital, Trabzon, Turkey

**Keywords:** Pilonidal disease, Staging, Classification, Limberg flap, Bascom operation

## Abstract

**Background:**

Although there are many therapeutic options to manage patients with sacrococcygeal pilonidal sinus disease, there remains controversy over a gold standard method for treating such patients. Most studies regarding sacrococcygeal pilonidal sinus, collected patients in a single pool, and single modality was performed to all patients so far. Staging according to the progressive nature of disease and comparisons of stage-based treatment approaches are yet to be conducted. This study aimed to define a staging system and to evaluate outcomes with the use of stage-based treatment approach.

**Methods:**

The collected data of patients who underwent surgery for the treatment of pilonidal sinus disease prior to June 2011 were analyzed. Following this analysis, a staging system was defined based on morphological extent of disease (stage I to stage IV for primary disease, and stage R for recurrent disease). Specific surgical technique was used for each stage. Between June 2011 and December 2014, 367 patients were operated based on proposed staging system and treatment algorithm. Demographics, perioperative data, short-term and long-term outcomes were evaluated according to the disease stage.

**Results:**

For all patients, the median length of hospital stay was 1 (range, 0–4) day. Primary healing without any wound complications was achieved in 320 (87.2 %) patients. The median time to functional recovery was 10 (range, 2–35) days and for wound healing was 12 (range, 10–55) days. Disease recurrence was identified in six (1.6 %) patients within the median follow-up period of 29 (range, 5–47) months. The outcomes of each stage were evaluated separately.

**Conclusions:**

We believe that the proposed staging system and stage-based treatment approach, which need further validation, will have an efficacy in the treatment of chronic pilonidal sinus disease and will contribute to the development of more appropriate individualized management approaches. Moreover, the use of this staging system will likely facilitate sharing and comparing more specific clinical data from future studies.

**Trial registration:**

NCT02712970 (ClinicalTrials.gov/09.03.2016)

## Background

Sacrococcygeal pilonidal sinus disease (PSD) is a common condition that predominantly affects young adults [1]. Although it is clinically asymptomatic in some cases, PSD may also present as a chronic, complicated disease, characterized by multiple sinus tracts, leading to severe impairment of patient quality of life. The heterogeneous nature of PSD presentation has been associated with the progressive course of pilonidal sinus development [2]. During the period of chronic abscess and epithelial tube development from normal hair follicles, the disease may affect more than one follicle and lead to lateral fistulization outside the midline [3].

To date, several studies have been conducted to determine the most appropriate treatment strategy for PSD [4–6]. Despite the heterogeneous nature of the disease, in most of the previous studies, all patients were collected in a single pool and a single treatment protocol was performed to all patients without grouping the patients and without considering the extent of the disease severity. These studies have provided data regarding a number of outcomes in these heterogeneous patient groups, including healing time (range, 1–78 weeks), time to return to work (range, 2–77 days), length of hospital stay (range, 2–14 days), and recurrence rates (range, 0 %–21 %) [4, 5, 7–9]. The application of a single treatment approach to all included trial patients has the potential to be harmful because it may contradict the “less is more” concept, causing cases with previously limited disease to progress to larger tissue defects [10]. Although the previously recommended classification systems have categorized the disease into several groups, staging according to the risk of disease progression is yet to be achieved [11, 12]. Further, direct comparisons of stage-based treatment approaches are yet to be conducted.

In this study, we aimed to propose a staging system in accordance with the progressive nature of pilonidal sinus disease. Moreover, we presented the results of patients treated with stage-based treatment approach that was suggested for each stage.

## Methods

This study was conducted at the Trabzon Kanuni Training and Research Hospital, a high-volume center for PSD and ethical approval was received from the Institutional Ethics Committee. The disease stages were determined from the database of patients who underwent surgery for the treatment of PSD prior to June 2011. Demographic data, preoperative and operative photographs, preoperative ultrasonographic images if done, and histology reports of excised specimens were analyzed. Following this analysis, a staging system (Stage I–IV) was defined as follows by using morphological extent of the disease:

- Stage I: Single pit in the midline, no lateral extension
- Stage II: >1 pits in the midline, no lateral extension
  - Stage IIa: 2–3 pits in the midline
  - Stage IIb: >3 pits in the midline
- Stage III: Midline pit/pits plus lateral extension in one direction
- Stage IV: Midline pit/pits plus lateral extension in both directions
- (Stage R: Recurrent PSD following any type of treatment)

**Fig. 1:**
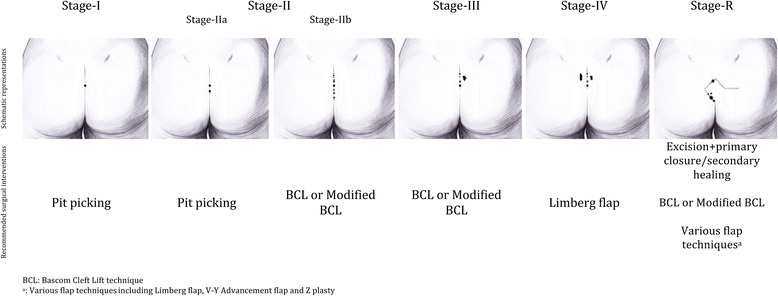
The schematic representations of the proposed staging groups and recommended surgical interventions for each stage

PSD which identified incidentally and which presented with acute abscesses were not included to the staging system. Moreover, patients with recurrent disease were included as a separate group, Stage-R. Specific surgical technique was used for each stage except for stage-R. The technique was decided based on the potential defect size following the excision of the diseased tissue. According to our algorithm, “pit-picking” technique was performed under local anesthesia on an outpatient basis in stage I and stage IIa patients [13]. In pit-picking technique, midline pits were excised removing a minimal amount of tissue (with a margin of skin of <1 mm). Incision of 1-2 cm in length was performed parallel to the most convenient side of the midline to be curetted of the chronic abscess cavity. All infected granulation tissue and hair were removed. After establishing hemostasis, the area of the excised midline pits was approximated by absorbable sutures. No specific postoperative wound care was given. For stage IIb and stage III patients, the Bascom Cleft Lift (BCL)/modified Bascom Cleft Lift (MBCL) techniques were performed [14, 15]. For stage IV patients, the rhomboid excision with the Limberg flap technique was used [16]. The BCL/MBCL/Limberg flap techniques that were described previously in detail, were performed under spinal anesthesia. The schematic representations of the proposed staging groups and surgical interventions that we recommend for each stage are shown in Fig. 1.

We prospectively collected data on patients undergoing surgery consecutively between June 2011 and December 2014. Surgical treatment was only recommended to symptomatic patients with the complaints of discharge or history of abscess drainage. Patients younger than 18 years of age and patients who were treated without the use of the suggested algorithm (operated by other surgical teams) were excluded from the analysis. Proposed algorithm was described in detail to all patients and written informed consent were obtained from patients. Prophylactic, preoperative antibiotics were administered to all patients. Drains were used according to the surgeon’s preference. Data regarding patient demographics, duration of symptoms, previous treatments, operative data, length of hospital stay, primary healing rate, functional recovery time, wound healing time, complications, and recurrence for 367 patients were analyzed.

Follow-up visits were on postoperative days 3, 10, and 30 and every 6 months thereafter by either follow-up visits or by phone. All patients were examined by a surgeon during follow-up period. Complications were classified as infection (superficial or deep), collection (seroma or hematoma), wound dehiscence (partial or complete), or anesthesia-related complications. All surgical site complications were recorded, and patients with prolonged healing were regularly examined until complete healing was achieved. Primary healing was defined as no breakdown of the wound (complication-free healing) at any point along its length. The patients were asked when they felt comfortable about starting their daily activities and the interval between surgery and return to daily activities was defined as functional recovery time. Recurrence was used when symptoms of the disease recurred some time after complete wound healing.

All parametric data values were presented as mean ± standard deviation and non-parametric data values were presented as median (range).

## Results

**Fig. 2:**
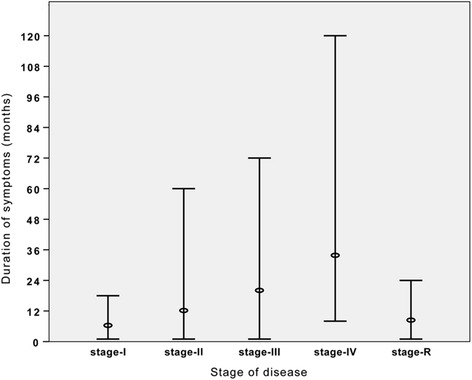
Median values of the symptom duration for each stage

The majority of the 367 patients who underwent surgery according to our proposed staging system presented with stage II disease (43.3 %). 71 patients (19.4 %) were classified as stage-I and all were operated because of the symptoms such as bleeding or purulent discharge even they have only one midline pit. No patient required additional intervention other than the recommended techniques in Fig. 1. Of the 367 included patients, 273 (74 %) were male and 94 (26 %) were female. The median age was 22 (range, 18–47) years, and the median body mass index was 23.6 (range, 18.2–41.6). The median duration of symptoms was 12 (range, 1–120) months, and 39 (10.6 %) patients had a history of abscess drainage. The duration of symptoms for each stage are shown in Fig. 2.

The median operative duration was 28 (range, 6–48) min. Surgical drains were used in 93 (25.3 %) patients, and the median time for drain removal was 1 (range, 1–3) day. The median length of hospital stay was 1 (range, 0–4) day. Demographic and perioperative patient data according to the disease stages are presented in Table 1.

**Table 1:** Demographics and perioperative outcomes according to the disease stages

		All (n:367)	Stage-I (n:71)	Stage-II (n:159)	Stage 2a (n:31) Stage 2b (n:128)	Stage-III (n:78)	Stage-IV (n:19)	Stage-R (n:40)
Age (years)		22 (18–47)	23 (18–47)	22 (18–45)	2a: 19 (18–26) 2b: 22 (18–45)	23 (18–45)	22 (18–24)	25 (18–46)
Sex								
	Male	273 (74.4 %)	57 (80.3 %)	117 (73.6 %)	2a: 19 (61 %) 2b: 98 (77 %)	52 (66.7 %)	19 (100 %)	28 (70 %)
	Female	94 (25.6 %)	14 (19.7 %)	42 (26.4 %)	2a: 12 (39 %) 2b: (23 %)	26 (33.3 %)	-	12 (30 %)
Body mass index (kg/m^2^)		23.6 (18.2–41.6)	24.2 (19.8–32)	24.9 (18.2–41.6)	2a: 25 (21.5–29.4) 2b: 24 (18.2–41.6)	25.4 (21.5–41.6)	25.1 (22.5–25.8)	26.5 (19.3–36.2)
Duration of symptoms (months)		12 (1–120)	5 (1–18)	12 (1–60)	2a: 6 (1–24) 2b: 12 (1–60)	12 (1–72)	23 (8–120)	6 (1–24)
Presence of abscess drainage history (n)		39 (10.6 %)	2 (2.8 %)	6 (3.8 %)	2a: 1 (3 %) 2b: 5 (4 %)	22 (28.2 %)	5 (26.3 %)	4 (10 %)
Operative duration (minutes)		28 (6–48)	8 (6–11)	28 (6–47)	2a: 8 (6–9) 2b: 30 (21–47)	29 (20–47)	44 (38–45)	33 (26–48)
Drain usage (n)		93 (25.3 %)	0	19 (11 %)	2a:0 2b: 19 (15 %)	28 (35.9 %)	19 (100 %)	27 (67.5 %)
Drain removal time (days)		1 (1–3)	0	1 (1–2)	2a: 0 2b: 1 (1–2)	1 (1–3)	1 (1–3)	1 (1–3)
Hospital stay (days)		1 (0–4)	0	1 (0–3)	2a:0 2b: 1 (1–3)	1 (1–4)	2 (1–4)	1 (1–3)

Primary healing without any wound complications was achieved in 320 (87.2 %) patients. Infection was observed in 20 (5.4 %, all superficial) patients, seroma in 28 (7.6 %), and partial dehiscence in 14 (3.8 %). The median time to functional recovery was 10 (range, 2–35) days and for wound healing was 12 (range, 10–55) days. In patients with complicated wounds, the median time for functional recovery and complete wound healing were 12 days and 18 days, respectively. Disease recurrence was identified in six (1.6 %) patients within the median follow-up period of 29 (range, 5–47) months. The short-term and long-term patient outcomes according to disease stages are presented in Table 2.

**Table 2:** Short-term and long-term outcomes according to disease stage

		All (n:367)	Stage-I (n:71)	Stage-II (n:159)	Stage 2a (n:31) Stage 2b (n:128)	Stage-III (n:78)	Stage-IV (n:19)	Stage-R (n:40)
Anesthesia complication (n)		9 (2.5 %)	0	3 (1.9 %)	2a: 0 2b: 3 (2.3 %)	4 (5.1 %)	2 (10.5 %)	0
Wound complications (n)								
	Infection	20 (5.4 %)	0	10 (6.3)	2a: 2 (6.5 %) 2b: 8 (6.3 %)	6 (7.7 %)	2 (10.5 %)	2 (5 %)
	Collection	28 (7.6 %)	2 (2.8 %)	7 (4.4 %)	2a: 3 (9.7 %) 2b: 4 (3.1 %)	11 (14.1 %)	2 (10.5 %)	6 (15 %)
	Dehiscence	14 (3.8 %)	0	4 (2.5 %)	2a:0 2b: 4 (3.1 %)	5 (6.4 %)	1 (5.3 %)	4 (10 %)
Primary healing (n)		320 (87.2 %)	69 (97.2 %)	143 (89.9 %)	2a: 27 (87.4 %) 2b: 116 (90.6 %)	62 (79.5 %)	16 (84.2 %)	30 (75 %)
Functional recovery time (days)		10 (2–35)	4 (2–7)	10 (2–35)	2a: 4 (2–7) 2b: 12 (7–35)	12 (7–25)	12 (10–14)	12 (8–22)
Wound healing time (days)		12 (10–55)	20 (12–35)	12 (10–55)	2a: 22 (12–30) 2b: 10 (10–35)	12 (10–40)	14 (12–20)	12 (10–22)
Recurrence (n)		6 (1.6 %)	0	1 (0.6)	2a: 1 (3.2 %) 2b: 0	2 (2.6 %)	1 (5.3 %)	2 (5 %)

## Discussion

In the present study, cases of PSD which is a progressive disease were classified according to the disease stage, and the patient outcomes following the use of a surgical treatment approach based on the disease stage were evaluated. Favorable primary healing rates and earlier functional recovery with acceptable morbidity was obtained for all disease stages, particularly in cases of early disease stages where limited surgery was performed. While stage II was the most frequent presentation type in the present study, extensive disease, defined as stage IV, was observed in only 5 % of the included patients. Moreover, a correlation between symptom duration and disease stages was determined by this study.

Clinical trials for PSD aim to identify simple and low-cost treatment approaches that require short hospital stays, decreased use of dressings, earlier recovery, and minimal impact on patient quality of life [17, 18]. However, despite studies being conducted for over a century, a gold standard technique for the treatment of PSD is yet to be defined. We believe the underlying cause of this failure to define the most appropriate treatment technique as the heterogeneous nature of the disease. Attempts to treat such heterogeneous clinical conditions with a single technique are unlikely to be the optimal approach. Therefore, the development of a PSD staging system is essential for decision-making processes regarding the management of this disease. Disease staging allows the use of more specific treatment strategies and estimation of prognosis. Further, it facilitates data sharing among investigators, thus enabling comparison of the results, quality, and cost of studies and may allow clinical studies to be performed in more specific patient groups. In the present study, four stages of chronic PSD were defined according to the degree of disease progression. The developed staging system informed the application of differing treatment modalities for different disease stages, with variable clinical outcomes obtained for each disease stage. For early disease stages, early recovery was obtained with patients returning to work within a few days. However, no increase in recovery time was observed in patients with late disease stages because of the extent of surgery and an increased incidence of wound complications. The comparisons of outcomes between disease stages were not performed. We believe that short-term and long-term outcomes should be evaluated separately according to a stage-based approach. The use of this disease staging system will likely facilitate sharing and comparing more specific clinical data from future studies, thus contributing to the development of individualized patient management strategies.

Unlike primary PSD, it is not feasible to determine disease severity or stage in case of recurrent PSD by evaluating the initial appearance of the gluteal area [19, 20]. Many factors, including morphological changes resulting from previous surgical procedures, time since previous surgery, development of new disease whether over previous surgical incision or not, and distinguishing recurrence and unhealed wounds, have an effect on the choice of treatment for recurrent disease. Therefore, it is unlikely that all recurrent cases can be appropriately managed with a single method of surgical intervention. Thus, we believe that a recurrent disease requires an individualized classification system. In the presented staging system, recurrent cases were classified as a separate group and were treated with a range of techniques. While asymptomatic disease is generally found incidentally, prophylactic surgery is universally considered to be of no benefit and we, therefore, chose not to include asymptomatic disease in our staging system [21]. Furthermore, acute abscess formation may develop at any stage of PSD and does not require the use of specific treatment approaches [22]. Therefore, we limited the developed staging system to primary chronic symptomatic disease only.

Stage I and stage II PSD are generally the earliest forms of symptomatic disease to present. While most cases of PSD begin as a single-pit disease arising from a single follicle, inflammation develops in other follicles over time because of the ongoing effect of predisposing factors previously described by Karydakis and Bascom [23–25]. At later stages, the involved follicles fistulize either unilaterally or bilaterally to areas outside the midline. Although the majority of surgical/non-surgical treatment methods can be used for all stages of the disease, various clinical experiences and habits among physicians have led to the development of a number of treatment approaches for PSD [26, 27]. Based on our previously published experiences, BCL/MBCL have become the preferred method for stage II/III disease in our practice [15, 28]. However, because this is an aggressive approach for stage I disease, we prefer the less invasive method, “pit-picking,” which can be performed under local anesthesia on an outpatient basis in cases of stage I disease. Further, we favor the “rhomboid excision with Limberg flap” technique in cases of stage IV disease, which enables wider tissue excision bilaterally and has favorable clinical outcomes [29]. In the developed algorithm, the extent of disease in stage II patients is determined by the number of pits located in the midline. Although it is possible to perform the pit-picking technique in patients with greater numbers of pits (up to 10), we limited the use of this technique in the present algorithm to patients with up to three pits because of a relatively high recurrence and reoperation rates of the pit-picking technique when used in all patients, regardless of disease extent [13, 30–33]. We therefore reserved the use of BCL/MBCL techniques for patients with greater numbers of pits. We believe this limitation enabled the low recurrence rates in patients that underwent pit-picking in the current study. While there was no recurrence in stage I patients, recurrence occurred in one stage IIa patient who had an uneventful postoperative period before the development of a new pit eight months after the pit-picking procedure. The higher rates of recurrence observed in patients with more than one pit supports our decision not to include patients with 1–3 pits in stage I and to divide stage II into two distinct subgroups. When determining the appropriate management for PSD, the risk/benefit ratio of the preferred modality regarding the speed of recovery and recurrence rates should be carefully considered an on individual basis.

To the best of our knowledge, the present study is the first staging system and stage-based treatment approach for PSD. However, this staging system has some limitations. Because PSD is present in a hidden anatomical area and can have an asymptomatic course, there are no validated methods for determining disease onset and progression. To overcome this, we used the data of patients who underwent surgical treatment prior to the beginning of the study to define disease stages. In the present study, we demonstrated a correlation between disease severity and duration of symptoms. We did not evaluate patient-related anthropometrics (body mass index, depth of intergluteal sulcus, thickness of fat tissue etc.) which probably have impact on the treatment outcomes. In future studies, anthropometrics should be included in the staging system; however, the addition of these factors could negatively affect the application of the staging system by increasing the complexity and making the algorithm more difficult to remember. The lack of cost analysis and the lack of comparison of cosmetic outcomes are other limitations of the present study. Besides, we used potential defect size as a determining factor to select the surgical treatment method. However, we know that various methods could be used for the treatment of PSD. In the future, studies comparing various techniques including surgical or non-surgical treatment methods for each disease stage are required to determine the most appropriate strategy.

## Conclusions

Chronic symptomatic PSD is a progressive disease that mainly affects young adults and is a significant cause of work loss. The identification of a single treatment approach for PSD has proved to be challenging because of the heterogeneous nature of clinical presentations in cases of PSD. Therefore, a more feasible approach may be to identify strategies for “the best management” rather than “the best technique” in future clinical studies. While it would be possible to define various staging systems and algorithms, the presented staging system showed acceptable morbidity and recurrence rates for all disease stages. We believe that the proposed staging system and stage-based treatment approach, which need further validation, will have an efficacy in the treatment of chronic pilonidal sinus disease and will contribute to the development of more appropriate individualized management approaches. Moreover, the use of this staging system will likely facilitate sharing and comparing more specific clinical data from future studies.

## Availability of data and materials

The data supporting the conclusions of this article are included within the article, tables and figures.

## Abbreviations

BCL: bascom cleft lift
MBCL: modified bascom cleft lift
PSD: pilonidal sinus disease
